# Percutaneous tricuspid valve repair using MitraClip® for the treatment of severe tricuspid valve regurgitation in a patient with congenitally corrected transposition of the great arteries

**DOI:** 10.1007/s12471-016-0866-y

**Published:** 2016-07-13

**Authors:** J. P. van Melle, R. Schurer, M. Willemsen, E. S. Hoendermis, A. F. M. van den Heuvel

**Affiliations:** Department of Cardiology, Thoraxcenter, University Medical Center Groningen, University of Groningen, 30.001, 9700 RB Groningen, The Netherlands

**Fig. 1:**
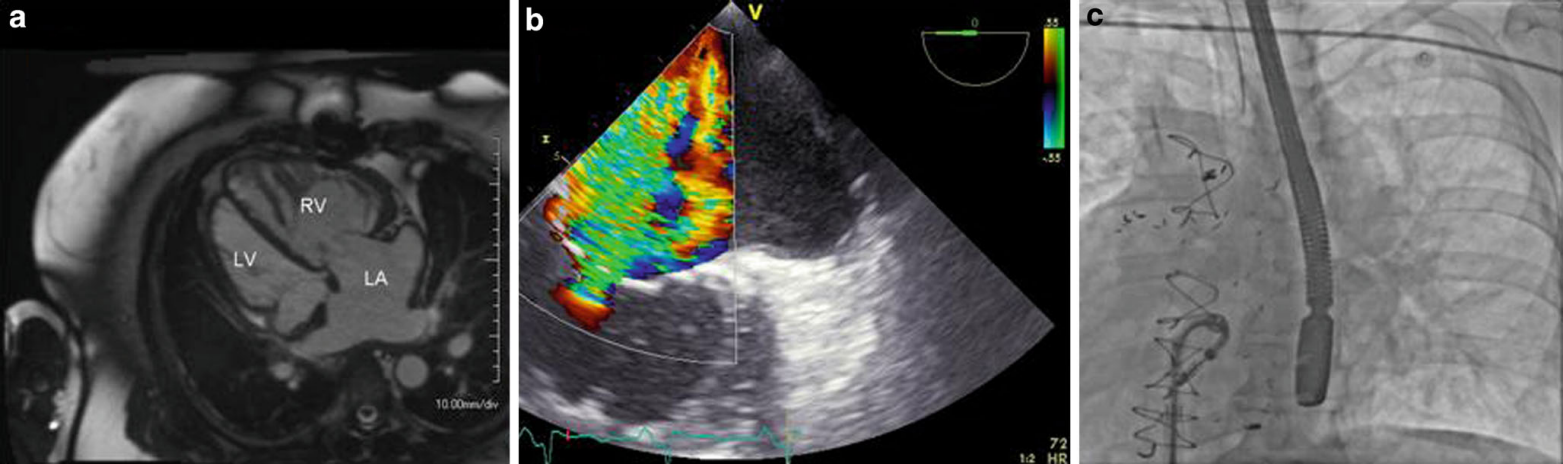
Magnetic resonance imaging showing a ccTGA diagnosis. The hypertrabeculated system ventricle is a morphological right ventricle (**a**). Echocardiographic images showing severe tricuspid regurgitation pre-MitraClip® (**b**). Fluoroscopy showing the delivery system and clip. Note the dextrocardia with apex to the right (**c**)

In selected patients, mitral valve repair using MitraClip® (Abbott, USA) is a relatively safe and well-tolerated treatment for significant mitral regurgitation [1–3]. We describe a 56-year-old female with congenitally corrected transposition of the great arteries (ccTGA) and dextrocardia (Fig. 1a) with recurrent episodes of heart failure caused by a combination of systemic (right) ventricular failure and tricuspid valve regurgitation (Fig. 1b). ccTGA is a rare congenital heart defect with discordance at both the atrioventricular and the ventriculoarterial level. In 20 % of the patients dextrocardia exists. Moderate to severe tricuspid valve regurgitation has a clear impact on cardiac prognosis [4]. We performed a percutaneous tricuspid valve repair using MitraClip® in 2014 (off-label use) (Fig. 1c). Six months after valve clipping, the tricuspid regurgitation was mild and there was an important reduction in heart failure symptoms. Mitral clipping may be feasible in selected patients with ccTGA. As far as we know, this is the first percutaneous tricuspid valve repair using MitraClip® in a patient with ccTGA and dextrocardia.
